# “Work with us… to make it more accessible”. What women with intellectual disabilities want from infant-feeding health resources: an exploratory study

**DOI:** 10.1186/s13006-023-00606-9

**Published:** 2023-12-08

**Authors:** Emma Douglass, Clare Johnson, Geraldine Lucas, Sally Dowling

**Affiliations:** 1 School of Health and Social Wellbeing, College of Health, Science and Society, University of the West of England, Glenside Campus, Blackberry Hill, Fishponds, Bristol, BS16 1DD UK; 2 School of Arts, College of Arts, Technology and Environment, University of the West of England Bristol, City Campus, Arnolfini, 6 Narrow Quay, Bristol, BS1 4QA UK; 3 Bristol Medical School, Faculty of Health Sciences, University of Bristol, 69 St Michael’s Hill, Bristol, BS2 8DZ UK

**Keywords:** Intellectual disabilities, Infant feeding, Resources, Breastfeeding, Visual methodologies, Accessible resources

## Abstract

**Background:**

More women with intellectual disabilities are becoming mothers but fewer are known to breastfeed compared with other women. Women with intellectual disabilities are entitled to accessible antenatal and infant feeding information, yet are rarely asked for their views on available resources. This article reports on the final stage of a UK project exploring how women with intellectual disabilities are supported to make infant feeding decisions. The wider project includes a scoping review and interviews with healthcare professionals, here we focus on the voices of the women themselves.

**Methods:**

Four women with an intellectual disability participated in a focus group where they were asked to give their views on the accessibility of currently available infant feeding resources and on alternative representations of infant feeding. All were interested in women’s health issues, including infant feeding. Photo-elicitation was used to gather views on videos, bespoke ‘Easy Read’ material and several alternative representations of infant feeding. A transcription of the discussion was thematically analysed whilst a critical visual analysis was undertaken of the women’s preferred images/resources. The study took place in Bristol, UK, during 2022.

**Results:**

Two themes were identified from the group discussion: ‘The desire for choice’ and ‘How easy is ‘Easy Read’?’ The desire for choice was expressed in terms through agreements and disagreements about preferred imagery, differing tastes, and reasons for these preferences. We identified a challenge to ‘Easy Read’ as a default standard and concerns that some forms of ‘Easy Read’ can confuse rather than inform. Critical visual analysis identified the importance of the story and social setting of the preferred infant feeding image.

**Conclusions:**

Findings suggest a need for a suite of resources, avoiding the one-size-fits-all approach, including people with an intellectual disability at every stage of the design and production process. Resources should recognise and embrace differences in terms of understanding, visual literacy and cultural taste, as well as being freely available to support women with intellectual disabilities to make informed infant feeding decisions. An accessible film was co-produced, to disseminate the findings from all three stages of the completed project.

## Background

This article reports on the final stage of a three-part project aimed at exploring how women with intellectual disabilities are supported to make infant feeding decisions. We were specifically interested in the acceptability of different visual images; the interdisciplinary nature of our research group (discussed below) facilitated our exploration of this. The first part of our project was a scoping review of published literature and resources, exploring how women with intellectual disabilities are supported to make infant feeding decisions with a specific focus on the visual images used [1]. We found few existing resources (with some of the resources hard to access), and limited evidence of how acceptable these are to women with intellectual disability.

The second part of our project explored how women with intellectual disabilities are supported from the perspectives of UK health professionals [2]. We interviewed seven health professionals about their experience in supporting women to make informed infant feeding decisions. Main themes found were ‘the importance of health professionals having unconditional, positive regard; the need for an individualised approach to supporting women to make infant-feeding decisions; and being part of the support network’ [2]. The final stage, outlined in this article, involved asking women with intellectual disabilities to attend a focus group to discuss the accessibility of currently available infant feeding resources. This article therefore concentrates on the views of the women themselves. Finally (as a separately funded project) we were able to make a film, co-produced with women with intellectual disabilities, to disseminate the findings from all three stages of the completed project.

This study was conducted in the UK, and whilst we acknowledge that this focus and setting may not represent the situation in all countries, we believe that our findings may be transferrable to other settings and of wider interest. In our earlier publications we chose to use the term ‘learning disabilities’ as this is currently used in England, where our research was conducted. Here, however, we have chosen to use ‘intellectual disabilities’ in recognition that this is used widely worldwide.

The number of women with an intellectual disability having children is increasing although exact numbers are difficult to report or are very country-specific [3]. Data from the UK’s Millennium Cohort Study suggests that 0.4% of the mothers in the cohort (n = 18,189) had an intellectual disability [4]. This population are likely to have poorer maternity experiences and health outcomes during pregnancy [5–9] and breastfeeding rates are lower than for other women [6, 10, 11].

To make an informed choice about infant feeding, information must be provided in an accessible way [1]. “Accessible” is defined by NHS England as information that can “be read or received and understood by the individual or group for which it is intended” [12]. Whilst this is a legal requirement in England [12, 13], a study by Homeyard and Patelarou [14] identified that accessible antenatal information is not routinely or consistently available. In a cross-sectional survey of sixty-five acute National Health Service (NHS) organisations, only seventeen were found to provide accessible information about breastfeeding, demonstrating a potential gap in provision for these women [14].

Our qualitative study exploring healthcare professionals’ perspectives of supporting women with intellectual disabilities in infant feeding decisions identified that several people are likely to be involved [2]. In this context, where multiple voices are in play – health professionals, social workers, family members, partners – women’s own voices are markedly absent within the published literature. This is reflective of wider literature focusing on the reproductive rights and choices of women with intellectual disabilities. Tilley et al. [15] and Earle et al. [16] highlight that the voices of women with intellectual disabilities have often been unheard, misunderstood or ignored within discussions about sexuality and reproductive rights, resulting in policy and practice not being supportive or representative of the population they serve. For future research exploring reproductive choices, Earle et al. advocate the use of an “inclusive co-research model” [16], acknowledging that this might take additional resources and time, but is imperative in improving services for women with intellectual disabilities. Our long-term intention is to recruit women with intellectual disabilities to the research team, as we have written elsewhere [1, 2]. Our aim for this phase was to gather the views of women with intellectual disabilities about infant feeding decision making. Using an exploratory approach, we asked:

i. What **supports** women with intellectual disabilities to make infant-feeding decisions?
ii. Which **images/media** are most effective in supporting decision-making in this area?

Throughout, we have attended to the use of visual imagery in existing resources. This has been enabled by the interdisciplinary nature of the research team, which includes specialisms in visual culture, intellectual disabilities nursing, midwifery, and public health. Our scoping review [1] revealed that there is a high degree of agreement within the identified literature about the importance of using visual images to communicate information about pregnancy, birth, and new motherhood - including infant feeding - to women with intellectual disabilities. However, we found little discussion of what constitutes accessible imagery, or which visual languages are effective, not least because women with intellectual disabilities are rarely asked. From both a methodological and political perspective it is crucial that the voices of women are heard. The purpose of this exploratory study was, therefore, to gain the views of women with intellectual disabilities on both the existing resources about infant feeding aimed at them and a set of alternative representations drawn from fine art, advertising, photography and social media.

Our research was conducted during the Covid-19 pandemic which produced many challenges for qualitative research, including with this participant group. Below, we reflect on some of these challenges and our learning about the research methods involved. These raise wider questions about trust and rapport in qualitative research and the need to be adaptive to both current social conditions and to the needs of specific participants. Our methods section (below) is therefore longer than is usual, reflecting our desire to share our learning in these areas.

## Methods

### Research design

We explored our research aims through a focus group, during which we used a photo elicitation method [17]. This uses photographs or other visual media either created by participants or, as in this case, selected by researchers to facilitate discussion. It involves showing participants a curated selection of images and asking them to comment. It is now a widely used qualitative research method, aiming to increase the depth of discussion by evoking emotions and memories as well as information [18]. Photo elicitation methods have been used previously in research with participants who have intellectual disabilities [19, 20]. It is a creative engagement method, which promotes inclusivity and active participation in the research process and is, therefore, well suited to working with this population.

The focus group allowed for discussion amongst participants and enabled us to identify the range of responses to the imagery presented as well as any consensus over the effectiveness, or otherwise, of specific images or image types. Focus groups are useful for collecting data on complex subjects that cannot be explored sufficiently with quantitative methods [21]. There is a risk of ‘group think’, but this was mitigated in our focus group by speaking to participants individually as they looked through the resources as well as in a group discussion.

### Recruitment

Arranging and facilitating the focus group during the Covid-19 pandemic posed some challenges. We used purposive sampling [22] to recruit women with an intellectual disability, who were interested in women’s health and willing to contribute to a discussion about infant feeding decision making. Initially, groups we knew were contacted and information disseminated via our networks. Some members of a local support group expressed discomfort at being involved, due to not yet having had the Covid vaccination. There was limited opportunity to introduce the project to potential participants and build trust and familiarity, as support groups were not running in-person at the time. Without the ability to introduce ourselves and the project to potential participants we could not build the rapport, trust and empathy that is crucial in qualitative research interviews [23, 24], so we decided to wait for lockdown restrictions to be lifted to enable an in-person event.

Holding an in-person focus group enabled us to draw effectively on our different disciplinary backgrounds, notably ED’s previous experience of interviewing women with intellectual disabilities and CJ’s experience of using visual materials as prompts for discussion. This caused considerable, but in our view necessary, delays to the project. We felt that viewing visual materials on screen would mediate interactions in ways that were not helpful to the research because it was important for participants to be able to handle the resources, show them to each other and physically rearrange the order. We also wanted to be able to provide support to participants if they found images challenging.

### Location

The choice of location for the focus group inevitably frames the research findings to some extent. Given that the focus group involved asking women to look at paintings and fine art photographs amongst other media forms, it was important to use a space that was neither saturated with expectations of cultural capital [25] nor resonant of a sterile waiting room. It was important participants felt psychologically safe to give their honest responses [26], as well as in the context of Covid ensuring that participants felt safe in the environment. We discussed locations for the focus group including university premises, community spaces and cultural institutions. We decided to facilitate the focus group at the university premises, where there was good ventilation and Covid guidelines at the time, such as the use of masks, were adhered to. In addition, this venue was accessible.

### Participants

Four women expressed interest in participating in the study and were given a copy of the Participant Information Sheet (PIS) or had conversations with ED about the study and opportunities to ask questions. Participants were between the ages of 34 and 53. All self-identified as having an intellectual disability. None of the women had a child or were pregnant at the time of the focus group. All were interested in women’s health issues, particularly for women with an intellectual disability, and were keen to participate in the focus group saying that infant feeding was an important topic. None of the women dropped out of the study. ED knew all the participants from previous work so another member of our research team, CJ, facilitated the focus group discussion.

### Choice of visual resources and mode of presentation

During the focus group we chose not to mount any materials on the walls, preferring to put A5 card-mounted resources and other visuals on tables so that they could be picked up and handled. This made it easy for participants to choose not to look, giving a degree of control that would have been lacking in a more exhibition-focussed form of display. In the room were four tables, the first for refreshments. On tables two and three we placed existing Easy Read resources on infant feeding retrieved through our scoping review [1] - pages from ‘All About Breastfeeding: for new mothers in North Wales’ [27], select pages from NHS Fife and Porter et al.’s ‘Pregnancy Support Pack’ [28] and the feeding section of an Easy Read book called ‘You and Your Baby, 0–1’ produced by CHANGE [29], a human rights organisation led by people with intellectual disabilities.

On table four was a laptop showing a series of thirteen short films on breastfeeding [30]. Our intention was to separate this from the Easy Read resources to avoid the audio-visual noise of the films impacting on the participants’ response to the printed resources. Here we also placed several alternative representations of infant feeding not used in existing resources. These included a colour breastfeeding photograph by birth photographer Leilani Rogers [31] (See Focus Group Image 1), a still from a Cow & Gate Ireland formula television advert [32], a painting by Artemisia Gentileschi called *Madonna and Child* [33], a ‘tree of life’ image by Samantha DeSanto [34], a photographic breastfeeding portrait by Ashlee Wells Jackson [35], an acrylic painting by Charla Maarschalk called *By Design* [36], and a colour bottle feeding photograph used by Babycenter.com [37]. We wanted to present a range of alternative visual representations of infant feeding produced using different media forms and evocative of different types of maternal femininity.

**Focus Group Image 1:**
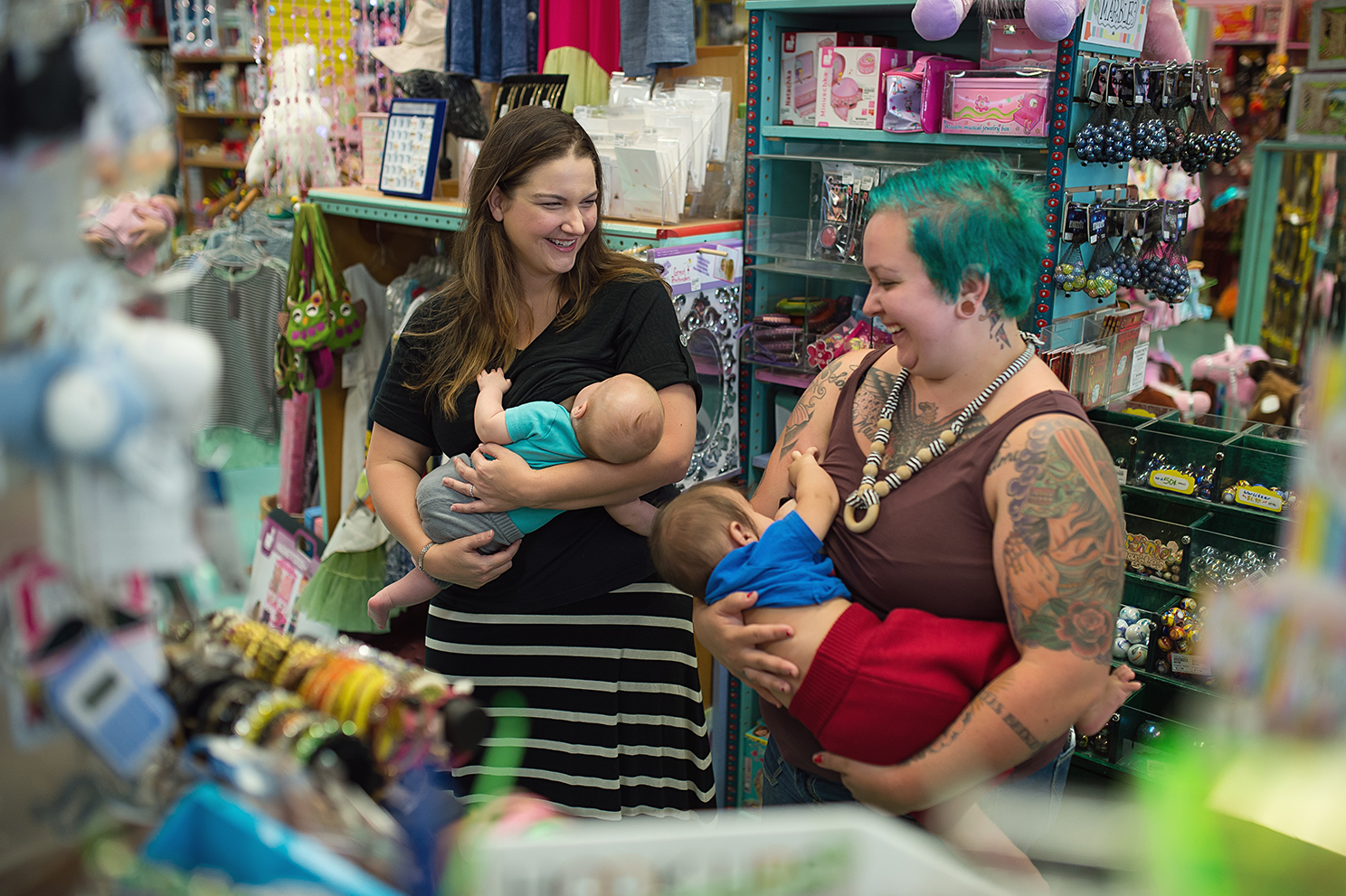
Terra toys_10 by Leilani Rogers

Three researchers were in attendance (ED, CJ and GL), enabling individual discussions regarding the intentions of the focus group and the consent process. Participants opted to choose their own pseudonyms. In the first hour participants viewed the range of infant feeding resources and were able to ask questions. After each woman had looked at the resources they were invited to discuss, as a group, which images or media resources they had found most helpful. This discussion lasted an hour, in which CJ asked questions (Table 1). ED clarified we had understood and captured each participant’s perspective as on occasions people spoke over each other or discussed alternative points. Participants were also asked to reflect on other important factors which could influence potential infant feeding decisions. The focus group was audio-recorded with participant consent. The data were stored in a secure OneDrive folder and subsequently transcribed in line with agreed ethics processes.

**Table 1:** Focus group topic guide

Focus group 1st hour to include: • Introductions and refreshments • Explanation of process including recording consent • Talking through Accessible Research Information Sheet • Explaining use of visual images (e.g. no right or wrong answers) and context of questions • Time for participants to look at range of resources / images on different tables.	
Opening question (approx. 5 min)	Do you find it helpful when something is explained using pictures instead of only using writing?
	or
	Do you like it when people explain things using pictures e.g., at the doctors?
Introductory questions (approx. 10 min)	What are your thoughts about becoming/being a mother? Which of the pictures do you like, and which do you not like? Can you tell us why?
	What is happening in this photo/drawing/painting in your opinion?
Key questions (approx. 20 min)	How do you feel about feeding a baby (breastfeeding and/or bottle feeding)?
	Do these pictures give you any information about how to look after a baby? What kind of information would you like about how to feed a baby? Are some of the pictures easier to understand than others? How do they make you feel? [In relation to one of two specific images.] Can you imagine being the woman in the picture? [We will be trying to find out if participants identify with any of the representations of maternity and if they feel excluded from any of them – how we do this will depend on the individuals who participate in the study.]
Ending question (approx. 10 min)	Is there anything you want to say about the pictures that we haven’t talked about?
	Is there anything else you want to say about the kind of information you would like about looking after a baby?

### Data analysis

We undertook a reflexive thematic analysis [38] of the focus group discussion data and a visual analysis [39] of key images of infant feeding shown to our participants. Rose’s framework for critically researching visual materials involves distinguishing between three sites: the site of production of the image (the way it is made), the site of the image itself, and the site of consumption or viewing by an audience. Each site is a point at which the meanings of an image can be generated. Within each site Rose considers three modalities: technological, compositional and social. These are not equally relevant to all visual images, so analysis involves making judgments about which are significant given the nature of the image. Rose argues that understanding these factors contributes to a critically engaged analysis of the visual image and that disagreements over the meaning of images are often disputes about which of these sites and modalities are more significant [39]. Braun and Clarke’s 6-stage iterative approach to thematic analysis [38] was used to analyse the focus group discussion. ED initially coded the transcript discussion, resulting in forty-four initial semantic codes. Through a process of discussion and further analysis, two themes were identified.

### Ethical considerations

#### Ethical approval

was granted by our University Faculty Research Ethics Committee. ED has expertise in working with people with intellectual disabilities, as a healthcare professional, healthcare educator and in relation to her doctoral studies. We drew on her experience in recruitment and gaining consent with this population; this was careful and extended. We viewed seeking consent to participate as a process [40], first sought at the beginning of the focus group, when each participant spent time with a researcher talking through the PIS and consent form. To ensure informed consent, this was also confirmed at the end of the focus group, after the participants knew what had been discussed.

## Results

The findings from the focus group are outlined here in two ways. Firstly, the thematic analysis is discussed in relation to the two themes identified – ‘The desire for choice’ and ‘How easy is Easy Read?’. This is followed by a critical visual analysis. Both are illustrated with quotes from participants.

Our participants were happy to look and comment on all the resources/images on display. At the end of the discussion, each reported to have enjoyed participating in the discussion, expressing interest in being involved in further research about infant feeding decision making.

### Thematic analysis

#### Theme 1: the desire for choice - “…ask, work with us… to make it more accessible” (Candy)

This first theme focuses on the women knowing what they liked and did not like, along with the reasons for their preferences. Whilst there was agreement about some of the images/resources we showed, the women had different ideas and disagreed with each other about others. For example, with the “tree of life” images, Summer liked the image, saying that it represented the baby growing but Lavender said that the image was confusing as it looked like a tree coming out from the breast. It was clear that choice was important, including having the relevant information to make the choice. Participants repeatedly identified that not all infant feeding options were shown or explained, identifying gaps within the resources, potentially limiting individual choice:

*“It also doesn’t show you the different ways you can feed the baby” (Summer)*.

Videos were discussed as being accessible, whilst the moving image shows you *what* to do, the audio explains *how* to do it:

*“talking things through” (Candy).*

Both together – the moving image and the audio – were found to be helpful in making the information (which might be complex) easier to understand. A key aspect for the preference of videos was the story that the moving image and audio portrayed:

*“Oh audio, yeah. Audio or video would be good. (…) Yeah. Someone telling a story.” (Lavender)*.

The value placed on watching and listening to a story was in conveying information in a way that was understandable, engaging, and relevant. These three aspects made video resources accessible to participants. Stories, however, could also be conveyed in photographs and images. When discussing a photograph of two women breastfeeding their babies whilst talking to each other in a public area (which all participants liked), one participant refers to the story behind the image:

*“Um, oh yeah, I liked that. It’s like, yeah, a story, as Summer said, you can think of a story between them, talking about babies and things” (Lavender)*.

Videos, particularly audio, were considered to be helpful in making information accessible, however participants felt strongly that people with an intellectual disability should be represented in these resources:

*“but they were all done with people without learning difficulties” (Candy)*.

This perceived under-representation of people with intellectual disabilities was mentioned by different participants, with a core belief that people with an intellectual disability should be involved in the development and execution of accessible resources.

#### Theme 2: how easy is ‘Easy Read’? “Like you have got cancer” (Lavender)

The second theme relates to the accessibility of the resources that have been specifically designed and developed for people with an intellectual disability. Whilst some aspects of some of the resources were helpful (for example, sequencing information in a step-by-step format), other aspects were bewildering, provoking discussion. The word ‘confusing’ was used frequently with regards to the use of line drawings, with participants struggling to understand what some were portraying. They questioned resources such as NHS Fife and Porter et al [28], which uses a mixture of line drawings and photographs:

*“The diagram ones aren’t that, um, clear as the actual picture (…) Why is there just one random picture in a diagram? (…) Why are you putting them (line drawings and photographs) together?” (Kacie)*.

*“I find it (line drawings) really inaccessible.” (Candy)*.

One part of a resource [27], which was trying to communicate the cost savings of breastfeeding was found to be particularly difficult to understand,

*“Because there is cost on the picture and also the purse picture is confusing as well. I don’t know what… the clock and the pig bank. And the pig bank it says savings. I don’t understand” (Lavender)*.

Images alongside words, often used within Easy Read resources, were particularly confusing, highlighting the importance of checking understanding and careful consideration of how important information is portrayed. For example, in one Easy Read document [27]:

*“Like you have got cancer (…) It says cancer but it looks like a radish or something” (Lavender)*.

*“Yeah. It’s got cancer and you are breast feeding!” (Summer)*.

Rather than portraying the intended message that breastfeeding can reduce the risk of cancer, participants were confused as to how cancer was related to breastfeeding, signifying that unless the individual understands what the ‘image’ is meant to mean/convey, the meaning can be lost, or worse, misunderstood.

*“Why breast feeding is best for you. And it’s the image of a woman and it says, it’s got a picture of something, broccoli” (Lavender)*.

This highlights the importance of checking that images convey the intended message. In this instance, a resource that has been designed to promote breastfeeding as an infant-feeding option, was understood to be communicating that breastfeeding will cost money and might give you cancer. There was agreement within the focus group that unless the individual understands what the image represents, it might communicate the opposite message to that intended.

### Critical visual analysis

During the focus group, participants sometimes disagreed about which sites and modalities were important [39]. This was apparent when, for example, Kacie praised the Best Beginnings short films for their clarity (site of the image itself) whereas Summer focussed on the fact that the films were not made using actors with intellectual disabilities (a social decision made during production). However, our participants all returned several times to the photograph by Leilani Rogers [31] (see Focus Group Image 1). This is one of the alternative images we used, which differs markedly from the Easy Read format prevalent within existing resources on infant feeding. Here we apply Rose’s framework to the Leilani Rogers photograph to understand more about the image preferences of our participants, thinking about the site of production, the site of the image and the site of consumption/viewing by an audience [39].

The site of production: the *technological* modality of this image refers to its existence as a photograph. One of the most striking and contested aspects of photography is its apparent truthfulness, a seemingly objective trace of reality or evidence of how things are. This view is heavily contested within cultural theory [41]. The prevailing view is that photographs show us not what the world looks like, but what it looks like photographed. This is to understand the image as constructed using the *compositional* familiarity of a particular genre, which in this case is documentary photography. The *social* modality refers to the social and political relations that surround the image, in this instance the social and cultural context within which breastfeeding takes place. This includes the photographer’s motivation to create empowering images that celebrate motherhood and contrasts sharply with shaming discourses of breastfeeding women [42]. Infant feeding support is often seen as tricky, risking emotional reactions which can either undermine or support decisions, contributing to mothers feeling embarrassed or self-conscious [43, 44]. These issues may be more acute for women who may already feel judged, observed and who may not see themselves in the images they observe.

The site of the image: this includes the composition and formal elements of the image e.g. use of colour, point of view, pose, cropping, exchange of looks. Two of our participants commented on the sociality of the composition:

*“That’s quite nice, you feel like friends socialising. You are feeding your baby, but you are also chatting about what’s going on in life with your baby. And asking advice.” (Summer)*.

*“Um, oh yeah, I liked that. It’s like, yeah, a story, as Summer said, you can think of a story between them, talking about babies and things.” (Lavender)*.

The photograph depicts an interaction between women, not between one woman and a healthcare professional. There is a relay of looks in which one woman looks at the other who, in turn, looks at one of the babies feeding. None of the characters look at the camera/viewer who is positioned as if in the space but not part of the interaction, thus allowing an unencumbered gaze with no confrontation. This sets up a visual dynamic that can be imagined as conversation and advice not instruction and risk management. In this sense it differs substantially from the visual tone of Easy Read imagery, which uses information graphics and diagrams to attempt a universal language of instruction. The women are standing in what appears to be a colourful gift shop, smiling and sharing the experience of infant feeding. Breastfeeding is represented as enjoyable, sociable, convenient and public. None of our participants commented on the public space of breastfeeding represented in this photograph. What mattered more was the sociality of the experience.

The site of consumption/viewing by an audience: this refers to the social practices that structure viewing. To view the images in a focus group is quite different to the experience of viewing them in a healthcare centre or at home. In practice health professionals are under pressure and have limited time to ensure that critical information has been understood. In the focus group we had deliberately presented alternatives to Easy Read imagery as an invitation to engage participants in a discussion about other forms of address. In this context the Leilani Rogers photograph functioned as a refreshing antidote to the text/image combinations of Easy Read resources, which by comparison were understood as more challenging to make sense of. Rogers’ photograph is not intended to impart healthcare information, yet it communicated something positive about breastfeeding on a social and affective level.

## Discussion

These findings reflect the perspectives of a small group of women who shared an interest in women’s health issues and the accessibility of health-related information, including around infant feeding. None of the participants were or had been pregnant at the time of the focus group. Further work with women with experience of making infant-feeding decisions would be helpful. As mothers with learning disabilities are less likely to breastfeed [6, 10, 11], and little is known about the acceptability of infant-feeding resources and representation to this population [1], the findings from this project are the first to shed light on the acceptability of current resources.

### Critical engagement

Images of infant feeding can be challenging for any participant group because they resonate with potentially triggering issues such as embodiment, motherhood, privacy, intimacy, and bonding. However, our participants were all prepared to observe the existing resources and alternative visual materials carefully, asking questions for clarification. All joined in the discussion during which they expressed their views about individual images and resources with clarity and conviction.

One of our ambitions was to listen to the voices of women who are often excluded from discussions that effect their lives. The women in our focus group were keen to express their views about the materials, make comparisons between them, draw on their own understanding (if not experience) of infant feeding, and were prepared to disagree with each other about the efficacy of the materials. This points to the polysemic nature of any visual representation [45], its capacity to mean in different ways for different constituencies, and the importance of acknowledging contestation about what an image means. In short, the participants approached the materials with a critical eye.

### Visual confusion

The findings suggest that some of the existing Easy Read resources are visually confusing. This is in line with suggestions that the inclusion of pictures and text, often used within Easy Read resources, can be confusing [46]. The use of visual metaphor in the All About Breastfeeding pack, for example, was not only difficult to understand but led to participants misunderstanding the relationship between breastfeeding and cancer. Such misunderstandings were prevalent where a combination of photographs and stock graphics or line drawings had been used either within the same image or on the same page. These confusions led Candy to advocate repeatedly for resources to be co-produced with women who have intellectual disabilities and for representation of this population in the moving image resources such as breastfeeding videos. Our findings also point to the impossibility of a universal visual language and a tendency to assume that Easy Read is always the answer. This raises the question of how these resources are produced, by whom and in what circumstances. The site of production often excludes professional design input [39]. The reliance on Easy Read as a default option is understandable in this context but belies an underlying issue with under-resourcing of healthcare information for women with (or without) intellectual disabilities. Certainly, our participants expressed a preference for images that were representational, not abstract, and favoured photographic images with high production values. The mode of address in their preferred images was embodied and emotive rather than instructional.

### Maternal subjectivity

Our participants expressed their tastes and values, which were informed, as with any other consumer group, by previous experience. For Summer, this included her knowledge of having been tube-fed her mother’s milk as an infant and an appreciation of the labour involved in this process. Our participants empathised with mothers learning how to feed their babies, even imagining themselves trying to follow the instructions in the various resources, despite none of them having had this experience.

Their responses highlight the importance of a wider, more inclusive understanding of the maternal, which includes, but is not limited to, those who have given birth [47]. Women with intellectual disabilities have historically been absent from discourses of active motherhood. However, a move towards the ‘maternal’ extends the discussion beyond the biological birthing experience to explore a wider network of maternal practices. For our participants this included an understanding of the care and labour involved in feeding, and the importance of the social bonds between mothers as well as the bonds between mother and baby. This was demonstrated in their enjoyment of Leilani Rogers’ photograph and their articulation of a maternal connection aligned to friendship and conviviality rather than instruction and judgment. Once invited into this discussion, our participants had no hesitation in offering their views.

### Strengths and limitations

This study is, as far as we are aware, the only one of its kind - allowing the voices of women with intellectual disabilities to be heard on this important topic. Alongside the other phases of our overall study [1, 2] it offers an important contribution to improving health and wellbeing for women with intellectual disabilities and their children. The women who participated in this phase were all well-informed, enthusiastic about taking part and wanted to be involved in future projects. As we have identified elsewhere, the interdisciplinary nature of our team and the different perspectives we bring to bear on the topic is also a significant strength. Limitations include that we were only able to recruit four women to participate and that none of these women had given birth or breastfed. Nonetheless, their contributions are valuable, coming as they did from women who were well-informed. At the end of the focus group, participants asked us how we would make the findings of all parts of the research project accessible. This led to a lively discussion and the idea to co-produce an accessible film of the findings. We subsequently received funding from the UK Arts and Humanities Research Council (AHRC) to work in partnership with the MISFITS theatre group [48] to co-produce a short film to communicate our findings [49]. It is freely available to view via YouTube.

## Conclusion

This project used an exploratory approach, with photo elicitation, to effectively facilitate a discussion, in a focus group, about existing resources and alternative infant feeding images. The women we spoke to were all well informed and keen to talk about infant feeding, saying that this was an important issue for women with an intellectual disability. The findings from this study support the findings from our previous work [1, 2] suggesting a need for the development of a suite of resources and a resource ladder, or decision tree, which avoids the homogenisation of this population (the one-size-fits-all approach), recognising and embracing differences in terms of understanding, visual literacy and cultural taste. Our experience demonstrates the importance of attention to recruitment, consent and location when working with this population, as well as the value of including their voices in research findings.

## Data Availability

This was a small, exploratory study with a group of potentially vulnerable women. The data derived from the study are therefore not available for use by others.
